# Progress and Challenges for Implementation of the Common Market for Eastern and Southern Africa Policy on Biotechnology and Biosafety

**DOI:** 10.3389/fbioe.2015.00109

**Published:** 2015-07-30

**Authors:** Michael Waithaka, Getachew Belay, Miriam Kyotalimye, Margaret Karembu

**Affiliations:** ^1^Association for Strengthening Agricultural Research in Eastern and Central Africa (ASARECA), Entebbe, Uganda; ^2^Alliance for Commodity Trade in Eastern and Southern Africa, COMESA Secretariat, Lusaka, Zambia; ^3^International Service for the Acquisition of Agri-biotech Applications (ISAAA), Afri-Centre, Nairobi, Kenya

**Keywords:** biosafety, policy, biotechnology, GMO crops, commercial planting

## Abstract

In 2001, the Meeting of the COMESA Ministers of Agriculture raised concerns that proliferation of genetically modified organisms (GMOs) could impact significantly on trade and food security in the region. This triggered studies on a regional approach to biotechnology and biosafety policy in Eastern and Southern Africa. The studies and stakeholder consultations revealed that farm incomes would increase if they switched from conventional varieties of cotton and maize to genetically modified (GM) counterparts. Commercial risks associated with exports to GM sensitive destinations, e.g., EU were negligible. Intra-regional trade would be affected since exports of GM sensitive commodities, such as maize, cotton, and soya bean, mainly go to other African countries. These findings justified the need to consider a regional approach to biosafety and led to the drafting of a regional policy in 2009. The draft policies were discussed in regional and national workshops between 2010 and 2012 for wider ownership. The workshops involved key stakeholders including ministries of agriculture, trade, environment, national biosafety focal points, biosafety competent authorities, academia, seed traders, millers, the media, food relief agencies, the industry, civil society, competent authorities, and political opinion leaders. The COMESA Council of Ministers in February 2014 adopted the COMESA policy on biotechnology and biosafety that takes into account the sovereign right of each member state. Key provisions of the policy include recognition of the benefits and risks associated with GMOs; establishment of a regional-level biosafety risk-assessment system; national-level final decision, and capacity building assistance to member states. The policies are the first regional effort in Africa to develop a coordinated mechanism for handling biosafety issues related to GMO use. A regional approach to biotechnology and biosafety is expected to foster inter-country cooperation through the sharing of knowledge, expertise, experiences, and resources.

## Background

Agriculture remains the backbone of economic development in Africa. “Agriculture contributes approximately 35% of the continent’s GDP and accounts for 70% of its labor force” (Falck-Zepeda et al., 2013b). For many years, African agriculture has been characterized by low productivity arising from little use of inputs and reliance on rainfall. Although notable progress has been made with respect to increasing productivity and availing access to inputs, many challenges stand in the way. Climate variability is a new reality as witnessed in erratic rainfall patterns, droughts, and emergence of new diseases, e.g., maize lethal necrotic disease. Rapid population increases that hover around 3% per year, and shrinking arable land continue to constrain provision of food. Providing solutions to these and other challenges is further complicated by the highly heterogeneous nature of African agro-climates, soils, farming practices across regions and within countries.

Contemporary food price crises in 2007/2008 and 2010/2011 compounded the challenges. High and volatile food prices negatively impacted food security of the vulnerable and poor, and undermined the trade competiveness of countries. It showed that only a few countries were self sufficient in basic food staples. Governments in developing and emerging economies quickly responded, especially in 2007/2008, with a myriad of policy measures that included price controls on food, cash transfers, agricultural input subsidies, use of food grain stocks, export restrictions for grains, lower import tariffs, and increased export taxes [ASARECA (Association for Strengthening Agricultural Research in Eastern and Central Africa) (2008)].

While some of the policies provide important safety nets for the vulnerable, protectionist measures can undermine trade. Trade is particularly important in that it not only affects the availability of and access to food in the short-run but it also affects the pace of growth of the economy as a whole. A major lesson was that while the short-term policy responses employed were useful in addressing immediate concerns, they were inadequate because the high-food price volatility continues to date. Short-term measures should be augmented with long-term measures. Long-term measures are required to address sustained support to the development of improved technologies and their adoption as well as measures to harness the comparative advantage afforded by regional cooperation. Such measures include harnessing science and technology to address productivity issues, access to inputs and knowledge and information about new technologies in the wake of evolving challenges. These measures are enshrined in the Malabo Declaration on accelerated agricultural growth and transformation for shared prosperity and improved livelihoods shared by African countries [AU (African Union) (2014)]. This is not a new idea, but a recommitment to the Maputo declaration of 2003 where African countries pledged to increase funding to agriculture to at least 10% of GDP in 10 years. The countries also adopted the principles and values of the Comprehensive Africa Agriculture Development Program.

Further, countries in Africa have embraced regional integration as a means to ultimately achieving free trade in goods and services. Key to this is adoption of common frameworks and policies as well as sharing resources. However, regional integration can only solve some problems. For example, trade concerns can be addressed through improved regional integration where movement of food is eased. On the other hand, increasing productivity requires the development and use of more adapted high yielding varieties and in this instance regional integration has some limited scope.

## Concerns Around Biotechnology in the Midst of Scientific Optimism in Africa

One promising, yet controversial, technological innovation is modern biotechnology, which has both regional and national dimensions. Biotechnology tools, including genetically modified (GM) crops and other organisms, have produced valuable products that have been adopted by a large number of farmers globally including those in Argentina, Brazil, China, and India. Africa has not been left behind. In 2014, Africa continued to make progress with South Africa’s land under GM crop production at 2.7 million hectares, marginally lower than in previous seasons mainly due to drought (James, 2014). Sudan increased Bt cotton hectarage by almost 50%, while drought precluded a potentially higher hectarage than 0.5 million hectares in Burkina Faso (James, 2014). In addition, Cameroon, Egypt, Ghana, Kenya, Malawi, Nigeria, and Uganda are in advanced stages of confined field trials, which are the penultimate step prior to approval (James, 2014). Importantly, the Water Efficient Maize for Africa project is scheduled to deliver the first stacked biotech drought tolerant maize with insect control (Bt) in South Africa in 2017 (James, 2014).

The major concerns to adoption of GM crop technology have revolved around safety to humans and animals that would consume the end products, and plants and insects in the environments where the crops would be grown. In Africa, there is wide belief that GM crops are intended for use in the industrialized countries, and are hence inappropriate for agriculture as it is practiced in Africa (Falck-Zepeda et al., 2013a,b). Second, with respect to trade there are concerns that GM crops would replace conventional varieties and thereby make farmers dependent on private seed companies. Third, there are also concerns with existing capacities to undertake research and effectively monitor and evaluate GM products and their use in the continent (Mulwa et al., 2013). Finally, loss of export markets for specific crops to trade-sensitive countries has also been expressed. This is amplified by fears that crops approved in one country but not another, may find their way through porous borders and informal trade. This may not be far fetched given that COMESA countries trade a lot with European countries where the levels of caution and consumer skepticism are relatively high (Paarlberg, 2008a; Wafula et al., 2012). These fears stem from uncertainty over those who gain and those who lose from the technology, unforeseen consequences, time before any impacts are discovered and what would happen if there were any irreversible damages (Omamo and von Grebmer, 2005). These fears continue to persist in debates on GM crops. Scientists and regulators are aware of these concerns. They address them through lengthy and stringent biosafety regulatory phases and decision-making that builds on stepwise accumulation of information.

A major paradox in the COMESA region is that while the development of the regulatory frameworks has been rather slow, scientists have made significant progress in developing various GM crops, such as potato, wheat, cucumber, cotton, cassava, sweet potato, banana, sorghum, cowpea, and rice, which are lined up at various stages of trials (Table 1). On close inspection, the technology has moved beyond initial focus on weed and insect control to addressing drought tolerance, controlling bacterial and viral diseases, and nematode infestation, improving nitrogen-use efficiency and bio-fortification (Table 1).

**Table 1:** **Biotech/GM crops and traits that are at or beyond confined field trial stages (CFTs) in COMESA Member States**.

COMESA member state	Crop	Trait	Stage
Egypt	Maize	Insect resistant	Commercialized in 2008, planting suspended in 2012
	Potato		
	Wheat		
	Cucumber		
	Melon		
Kenya	Maize	Drought tolerance	CFT, fifth season
	Cotton	Insect resistant	CFT, third season
	Cassava	Mosaic disease	CFT, second season
		Brown streak virus	CFT, second season
		Vitamin-A enriched	CFT, second season
	Sweet potato	Virus disease	Application for CFT
	Sorghum	Bio fortified (ABS)	CFT, second season
	Banana		
Malawi	Cotton	Insect resistance and herbicide tolerance	CFT, second season
	Cowpea	Insect resistance	Application for CFT
Sudan	Cotton	Insect resistant	Commercialized, third year
Uganda	Maize	Drought tolerance	CFT, fifth season
		Insect resistance	CFT, first season
	Banana	Bacterial wilt resistance	CFT, second season
		Nutrition enhanced	
		Nematode resistance	CFT, second season
	Rice	Nitrogen-use efficient, drought tolerance	CFT, second season
	Cassava	Mosaic virus, brown streak virus	Multi-location trials
	Potato	Blight resistance	CFT, second season
	Sweet potato	Weevil resistance	CFT, second season

*Source: adapted from ACTESA (Alliance for Commodity Trade in Eastern and Southern Africa) (2015)*.

The optimism shown by scientists mirrors global trends where adoption of GM crops has been on a steep rise. In 2014, 7.1 million small scale farmers in China and 7.7 million in India elected to plant over 15 million hectares of Bt cotton because of the significant benefits it offers” (James, 2014). Similarly in 2014, “415,000 small scale farmers in the Philippines benefited from biotech maize. Biotech crop hectares were planted in 28 countries in 2014 and hectarage has increased more than 100-fold from 1.7 million hectares in 1996 to 181.5 million hectares in 2014 – a 6.3 million hectare increase compared to 5.0 million hectares in 2013 at an annual growth rate of between 3 and 4%” (James, 2014).

## COMESA: Toward Regional Economic Integration

Since the launch of the COMESA Free Trade area in the year 2000, there has been a steady increase in formal and informal intra-COMESA trade in agricultural products [COMESA (Common Market for Eastern and Southern Africa) (1994)] (Box 1). The provisions of Article 129 of the COMESA Treaty stipulate full cooperation in agricultural development, science and technology domains, to increase agricultural production and attain regional food security [COMESA (Common Market for Eastern and Southern Africa) (1994)]. Further Article 130(a) of the COMESA Treaty stipulates that “Member States undertake to co-operate in specific fields of agriculture, including the harmonization of agricultural policies of the Member States with a view to having a common agricultural policy”.

Box 1 **Facts about COMESA.**

The Common Market for Eastern and Southern Africa (COMESA) is the largest grouping and trading bloc in Africa in terms of area, population and number of Member States. COMESA evolved in 1994 from the predecessor the Preferential Trade Area for Eastern and Southern Africa, which had been established in 1982 with the aim of forming an economic community in the region. The Member States are: Burundi, Comoros, Democratic Republic of Congo, Djibouti, Egypt, Eritrea, Ethiopia, Kenya, Libya, Madagascar, Malawi, Mauritius, Rwanda, Seychelles, Sudan, Swaziland, Uganda, Zambia, and Zimbabwe. The 19 COMESA Member States represent over 440 million people, $32 billion/year in imports, and $82 billion/year in exports (Azzarri et al., 2014). The average growth rate of the gross domestic product was 6.6 % in 2013 (COMESA, 2014b). In 2014, the annual import bill was US$170 billion while the export bill was US$112 billion (COMESA, 2014b).

The design of COMESA aimed to remove the structural and institutional weaknesses in the Member States by strengthening collective action especially in pooling of resources to sustain development efforts. Since 2008, COMESA has been working together with the East African Community and the Southern Africa Development Community towards a Tripartite Free Trade Area Agreement that will bring together 26 Member States. This expanded market holds promise of benefits of economies of scale and complementarities.

One of the aims and objectives of the COMESA Treaty (Chapter 3, Article 3a) is to co-operate in the creation of an enabling environment for external, cross border, and domestic investment including the joint promotion of research and adaptation of science and technology for development [COMESA (Common Market for Eastern and Southern Africa) (1994)]. Chapter 18 of the Treaty encourages Member States to co-operate in the harmonization of agricultural policies of Member States with a view to having a common agricultural policy and the control of animal and plant diseases and pests [COMESA (Common Market for Eastern and Southern Africa) (1994)].

Development of the COMESA policy on biotechnology and biosafety was informed in part by the recognition of the potential for a regional framework to harness potential benefits of science and technology and prudent regulation. The policy seeks to ensure responsible governance of modern biotechnology in the COMESA region. This paper reconstructs the process from the development and eventual approval of the regional biotechnology and biosafety policy. We describe the key elements of the Policy and discuss the challenges and opportunities derivable from the policy. The paper ends by identifying key lessons for similar efforts in other regions.

## Problem Statement and Justification

Half of the projected increase in global populations by 2050 will occur in Sub-Saharan Africa. Population growth is expected to drive increases in demand for food, feed, and fiber. It is projected that agricultural food demand to 2050 will rise by 50% (Tilman et al., 2001) while demand for animal feeds will increase by 84% by 2020 (Delgado et al., 1999). To meet this demand, options for enhancing yields include expansion of the area under cropland at the expense of other ecosystems, increasing yields on existing croplands and or reallocating current agricultural production to more productive uses (Licker et al., 2010).

In the past two decades, cereal yields have shown consistent increase across Africa, from 1,159 kg/ha during 1990–1995 to 1,448 kg/ha during the 2003–2012 period (Bahiigwa et al., 2014). Cereal yields have increased most in eastern and southern Africa and least in northern Africa. The highest yields in the 2003–2012 period were reported in the COMESA region, with 1,780 kg/ha, followed by EAC with 1,627 kg/ha (Bahiigwa et al., 2014). Although, yields have gone up, the yield gap remains substantial. Globally, the yield gap for wheat and rice has been estimated at 36% and at 50% for maize. In Africa, the yield gap for maize is as high as 80% (Neumann et al., 2010).

Agricultural biotechnology stands out among the diverse options available that would significantly improve crop yields and household incomes in an environmentally sustainable way. Trends indicate that the global adoption of the technology is high and increasing. Developing countries mainly in Asia, Africa, and Latin America are among the leading adopters of the technology. “By 2013, biotech cotton in countries, such as China, India, Pakistan, Myanmar, Burkina Faso, and South Africa, had already made a significant contribution to the income of approximately 16.5 million poor farmers” (James, 2014). With these developments and the increased field trials currently being undertaken in many African countries, a regional approach to decision-making on genetically modified organisms (GMOs) has become a fundamental issue because of the trans-boundary nature of GMOs (Timpo, 2011). African countries can make bigger strides in this area by enacting functional, cost-effective and predictable biosafety frameworks while at the same time riding on the back of a regional framework to ease tensions between countries.

Many studies have documented benefits accruing to farmers in developing countries, who have adopted the technologies (Mbwika, 2006; Paarlberg, 2008a,b; Mulwa et al., 2013). For example in a study on Bt cotton welfare analysis for countries in the COMESA region, showed that every country gained in all scenarios that were considered (Mulwa et al., 2013). In the same study, the highest gains from adopting Bt cotton would accrue to Egypt, while Kenya had the least gains. The study showed that returns per hectare were similar in all countries in the sub-Saharan part of COMESA. “The distribution of gains varies between the different categories of players in the Bt cotton industry, with most of the gains accruing to producers and consumers while the least accrues to innovators of the technology” (Mulwa et al., 2013).

These results are supported by a recent meta-analysis (Klümper and Qaim, 2014), which confirms that “the average agronomic and economic benefits of GM crops are large and significant”. The same analysis further showed that “yield gains and pesticide reductions are larger for insect-resistant crops than for herbicide tolerant crops” (Klümper and Qaim, 2014). Scientists in Africa have been relentless in their claims that the continent stands to gain a lot from GM technology. This is supported by analysis that shows that yield and farmer profit gains were higher in developing countries than those achieved in developed countries (Klümper and Qaim, 2014). The same analysis shows an unusual finding with respect to yields. It shows that on average, GM technology had increased crop yields by 21%, from more effective pest control and thus lower crop damage and not due to higher genetic yield potential. Despite the fact that GM seeds are more expensive than non-GM seeds, the additional seed costs are compensated through savings in chemical and mechanical pest control. “GM crops reduced pesticide quantity by 37% and pesticide cost by 39%” (Klümper and Qaim, 2014). A study on the impacts to farm incomes in Egypt, Ethiopia, Kenya, Tanzania, Uganda, and Zambia found that average profit gains for GM-adopting farmers were 69%” (Paarlberg et al., 2006a). These studies support the notion that on average, adoption would be profitable. However, it should be appreciated that results based on average measures do not provide the true picture since they mask variability in many conditions, such as agro-climates, host cultivars, and farming practices (Falck-Zepeda et al., 2013a,b). This may be the reason many policymakers and farmers in Africa hesitate to fully embrace GM crops. They ask for African context specific information about the potential, benefits, costs, and safety of GM crops (Mulwa et al., 2013).

However, analyzing the biosafety of specific GMOs requires physical, human, and financial resources, which may be out of reach for many countries in the COMESA region. Given that the majority of COMESA Member States are yet to establish their national biosafety regulatory frameworks, this then strengthens the need for regional cooperation. A regional mechanism will assist countries to share information, resources, and expertise as they gradually build the necessary capacities for biosafety risk assessment and develop and implement effective national biosafety frameworks. This regional mechanism is supported by the Cartagena Protocol on Biosafety (CPB), which 17 out of the 19 COMESA countries are signatories to. Article 14 of the protocol provides that countries may enter into bilateral, regional, and multilateral agreements and arrangements to manage trans-boundary movement of GMOs [SCBP (Secretariat of the Convention on Biological Diversity) (2000)].

## Methodology

This paper uses a narrative approach to document and share knowledge on the “how” around the process to eventual adoption of the COMESA biosafety policy. Key sources of information and data include declarations of the relevant COMESA Ministerial meetings, project reports and publications, proceedings of national and regional stakeholder consultative fora on the policy and its communication strategy, minutes of the drafting process and related literature on biosafety issues within COMESA Member States. This information was used to construct the story on the process toward approval of the COMESA Biotechnology and Biosafety Policy and to develop perspectives on challenges, opportunities and lessons learnt.

## Results and Discussion

### Process to approval of the COMESA biotechnology and biosafety policy

In the years 2001 and 2002, countries in Southern Africa struggled with a severe drought where more than 15 million people faced starvation (Omamo and von Grebmer, 2005; Paarlberg, 2008a,b). This was not the first time this was happening. A similar situation happened in 1991 “as a result of poor weather, policy failures, and market failures” (Omamo and von Grebmer, 2005) Considering that most of the maize food aid would come from countries that had approved commercialization of GMOs, the debate on the safety of GM based food to humans and the environment surfaced. The reactions in 2002 were drastic. Zimbabwe, Mozambique, Lesotho, and Malawi took policy decisions that limited the import of food aid with GM content (Paarlberg, 2008a). They placed various restrictions on imports of un-milled GM yellow maize from the World Food Program, and Zambia refused all GM maize even if milled. Only Swaziland continued to accept un-milled GM maize without restriction as food aid through the World Food Program. These developments seem to have triggered action from the regional economic blocs. A meeting of the Southern African Development Community council of Ministers for Food, Agriculture and Natural Resources in July 2002 in Maputo, Mozambique noted, “the lack of a harmonized position on GMOs was creating serious operational problems in movement of food and non-food items” (Omamo and von Grebmer, 2005). The COMESA Council of Ministers meeting on November 29, 2002 in Lusaka Zambia endorsed the recommendation of the meeting of Ministers of Agriculture on November 4, 2002 in Kampala, Uganda, that “COMESA should develop a common position on GMO’s and other products of biotechnology” [COMESA (Common Market for Eastern and Southern Africa) (2002)]. This led to the development of the Project on Regional Approach to Biotechnology and Biosafety Policy in Eastern and Southern Africa (RABESA). In part, RABESA was to inform and support the move toward improved regional policy coordination (Wafula and Waithaka, 2006).

During the first phase of the RABESA project (2004–2007), studies were commissioned to analyze: (i) potential farm-income gains from the adoption of GM crops; (ii) the magnitude of commercial export risks associated with GM crops; and (iii) the impacts of delivery of emergency food aid with GM content in the COMESA region. The research findings were disseminated and discussed at national consultative meetings in COMESA Member States.

The study on farm-income gains projected that COMESA Member States could harness substantial benefits from the adoption of GM insect-resistant varieties of cotton and maize (Mbwika, 2006). The second study showed that use of Bt cotton for commercial planting might save cotton in developing countries from bollworm damage and provide farmers with higher levels of income (Paarlberg et al., 2006b). The control of bollworms is done through application of pesticides, which is a costly exercise in terms of the cost of pesticides, spray equipment, and labor (Mbwika, 2006).

With respect to trade-related implications of adopting GMOs in the COMESA region, the main conclusion was that inter-regional export losses associated with the adoption of GM crops in the COMESA region were negligible (Paarlberg et al., 2006a). Although COMESA countries depend heavily on the export of agricultural products to earn foreign exchange, the major exports were coffee, tea, sugar, horticulture, banana, and pyrethrum. None of these crops had been commercialized anywhere in GM form, meaning that there would be little or no GMO associated risk to agricultural export incomes (Paarlberg et al., 2006a). The food aid import policies study revealed that sub-Saharan Africa (SSA) was the largest recipient of emergency food aid globally, and COMESA countries received 85% of all emergency food aid to SSA. About 50% of the food aid arrives as donations from countries that are leading producers of GM crops, including USA and Canada (Paarlberg et al., 2006b).

These findings were presented in a regional workshop that brought together 40 key stakeholders from COMESA Member States that was held in Nairobi in 2006 (Wafula and Waithaka, 2006). The workshop considered the three policy harmonization options with respect to commercial planting of GMOs; commercial trade policy in GM products; and policy on access to emergency food aid with GM content. This was in line with the aspirations of the African Union conference of agriculture ministers in the same year, which recommended that the continental body set up mechanisms to identify commonalities and coordinate policies on biosafety and biotechnology [AU (African Union) (2006)]. With respect to commercial planting of GM crops the recommendation of the workshop was for countries to adopt a centralized regional assessment and national-level decision-making. The advantages of this approach were that it was standardized, transparent, cost–effective, and allowed sharing of resources, information, and expertise. The recommendation on commercial trade policy in GM products was for countries to get advice/information from a central regional clearing house, and retain decision-making at the national level. The advantages of this approach were the cost-effectiveness of regional-level assessment, cooperation in assessing trade issues, and sharing of information and capacities. The recommendation on access to emergency food aid with GM content was to have guidelines developed at regional level and national-level decision-making on a case-by-case basis. The advantages of this approach were that it would facilitate transit of food aid; facilitate provision of relief food, as well as timely humanitarian response.

These recommendations were presented, discussed, and endorsed at the fourth meeting of the COMESA Ministers of Agriculture held in Khartoum, Sudan, in March 2007 [COMESA (Common Market for Eastern and Southern Africa) (2007)]. The second phase of RABESA kicked off in 2008 with two additional partners. The International Service for the Acquisition of Agri-biotech Applications and the Alliance for Commodity Trade in Eastern and Southern Africa (a specialized Agency of COMESA). In response to the Ministerial directives, the RABESA team initiated the drafting of COMESA Regional Biosafety Policies and Guidelines in the three priority areas: commercial planting of GMOs, trade in GM products, and handling of emergency food aid with GM content. A biosafety roadmap and a communication strategy were also drafted to support the harmonization process. These documents were presented and discussed at regional and national consultative meetings to obtain feedback from Member States. The draft policies and guidelines were presented and discussed in a regional workshop that was held in April 2010 in Nairobi, Kenya.

The recommendations from the regional workshop were presented in the third Joint meeting of the COMESA Ministers of Agriculture, Environment and Natural Resources that was held in July 2010, in Lusaka, Zambia. The Ministers endorsed the biosafety roadmap and a communication strategy. They decided that the draft policies be subjected to further national consultative processes for wider ownership before they are considered by the COMESA policy organs. Following this decision, national consultative workshops were held in the COMESA countries between September 2010 and September 2011. Participants in the workshops were key stakeholders drawn from diverse institutions including ministries of agriculture, trade, environment, national biosafety focal points, biosafety competent authorities, seed traders, millers, the media, food relief agencies, the industry, civil society, competent authorities, and politicians/opinion leaders. A final regional validation workshop that brought together 40 participants, drawn from 15 out of the 19 Member States was held in May 2012 in Lusaka, Zambia. The participants endorsed the policies and recommended that they be combined into one.

**Figure 1:**
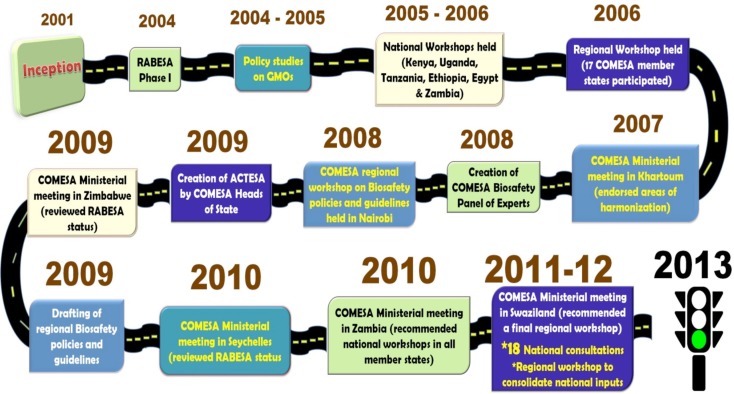
**Long road to the approval of the regional biosafety policy**.

The validated regional biotechnology and biosafety policy was presented, discussed, and approved at the fifth joint meeting of the Ministers of Agriculture, Environment, and Natural Resources that was held on 20th September 2013 in Addis Ababa, Ethiopia [COMESA (Common Market for Eastern and Southern Africa) (2014a)]. The policy was formally adopted by the 32nd meeting of COMESA Council of Ministers that was held on 24th February 2014, in Kinshasa, Democratic Republic of Congo. The COMESA biotechnology and biosafety policy is the first at the global level, coordinated mechanism for handling biosafety issues related to GMOs use at the regional level. The long and unwinding journey from the start of the RABESA project to the approval of the biosafety policy is depicted in Figure 1.

Two challenges stood in the way to the development of the COMESA biosafety policy. The first was that most COMESA Member States did not have functional national biosafety frameworks. Second, countries in Southern Africa had already challenged GM technology when they declined to receive food aid imports even when many people were faced with starvation. To date, these effects of these challenges are still being felt. In such a situation, then one would expect countries to have been anxious over talks of a regional policy, fearing that they would be ceding their sovereignty in decision-making at the national level. It is not surprising that concerns over sovereignty often threatened to derail the development of the regional policy. This was taken into consideration and the principle of national sovereignty is recognized as the cornerstone of the COMESA Policy.

Concerns over safety and long-term impacts of GM technologies still persist. For a start, the RABESA project generated evidence on potential impacts at farm level, on trade and with respect to food aid (Paarlberg et al., 2006a,b,c). Of the three areas, of study, the threat of losing export markets in major destinations, such as Europe, was a paramount issue in blocking adoption of GM crop. This debate continues to date despite evidence from several studies that have indicated that the magnitudes of risk are negligible. This is mainly because COMESA countries do not export to Europe any commodities that are currently available in GM form.

An unforeseen challenge occurred at the COMESA level. Prior to the year 2009, the Ministries of Agriculture handled all agricultural discussions. In 2009, the sittings of Ministries of Agriculture and those of the Ministries of Natural Resources and Environment were combined into what was thereafter referred to as joint Ministerial meetings. This was welcomed in that it implied that environmental concerns would also be considered alongside agricultural issues. The downside was that this slowed down the process for the biosafety policy because it necessitated more consultations with experts from the environment department who were hitherto not part of the GM debate.

When the second phase of RABESA started, it became imperative to have more consultations with the COMESA secretariat. This proved to be challenge and led to delays in getting some decisions made. This was overcome when the Alliance for Commodity Trade in Eastern and Southern Africa (ACTESA), specialized agency of COMESA, was assigned to lead the COMESA Biotechnology program in 2009. This shortened consultations and decision-making and speeded-up the progress thereafter.

### Key elements of the COMESA biotechnology and biosafety policy

The regional biotechnology and biosafety policy [COMESA (Common Market for Eastern and Southern Africa) (2014a)] was designed to provide guidance and facilitate decision-making on how to manage transboundary movement of GMOs across the porous borders safely and responsibly. It was envisaged that a centralized regional risk-assessment policy would allow COMESA countries to apply a harmonized approach to planting, trade, and handling of emergency aid of GM crops and mitigate the anticipated threat of disruption in intra-regional trade on products containing GMOs. The approach would help foster the goal of the COMESA Customs Union established in 2009 by the COMESA Heads of State to promote economic integration through unrestricted movement of goods, services, and people. Boxes 2 and 3 present excerpts from the policy [COMESA (Common Market for Eastern and Southern Africa) (2014a)] highlighting the objectives, exemptions and general provisions.

Box 2 **Objectives of the policy are to:**

- “provide COMESA Member States with a mechanism for scientific regional risk assessment of GMOs intended for commercial planting, trade and food aid in the COMESA region.”
- “provide a technical opinion about the biosafety of GMOs seeking commercial status in the COMESA region that can be used by individual countries to make decisions within their own national biosafety regulatory frameworks.”
- “provide a harmonized mechanism for decision-making involving commercial planting, trade of GMOs and food aid with GM content in the COMESA region.”
- “assist COMESA Member States share and build capacity in order to conduct scientific risk assessment and management.”
- “establish interactive regional information sharing mechanism on biosafety and biotechnology issues in the COMESA region.”

**Exemptions of the policy**

- “Socio-economic, cultural, liability and redress, labeling, and other country-specific considerations regarding GMOs will be handled at the national level in accordance with national laws and biosafety frameworks.”
- “Activities conducted with GMOs in the laboratories and confined field trials, leading up to commercial planting, will be handled at the national level in accordance with national biosafety frameworks.”
- “This policy does not imply changes in the rights and obligations of a Member State under existing international and other regional agreements.”
- “This policy does not impact or supersede other laws that apply to seeds, food, plants or animals irrespective of whether they are GMOs (such as general food safety legal requirements, seed variety registration, plant quarantine, among others).”
- “The policy specifies that all topics related to biosafety not specifically addressed within the Policy remain the responsibility of the national biosafety frameworks of Member States.”

Box 3 **General provisions on structure and procedures to implement the policy**

- “The COMESA Secretariat will establish a regional biosafety risk-assessment desk.”
- “The COMESA Secretariat will be responsible for administration and management of the regional risk-assessment activities (information storage, sharing and exchange) in the COMESA region and liaise with national, other regional and international bodies.” “National Competent Authorities will be the contact focal points, and in Member States without official competent authorities contact focal points will be identified.”
- “The COMESA Panel of Experts on Biotechnology and Biosafety (PoE) will be the main guiding body to formulate a risk-assessment “Opinion” on applications submitted and advice sought by Member States.”
- “The scientific risk assessments analyzed by the PoE will assess the GMO for possible risk to human and animal health, biodiversity, and the environment.”
- “The decision to approve or reject a GMO for commercial planting, based on the “Opinion” from the PoE rests on the sovereign decision of the individual COMESA Member State. COMESA, through the PoE shall prepare the detailed standard operating procedures, including dispute resolution, for each of the three policy areas; commercial planting of GMOs, trade in GMOs, and emergency food aid with GM content – in accordance with this Policy, and taking into consideration the national biosafety frameworks of Member States.”
- “COMESA will work with Member States to establish programs dedicated to creation of awareness on the existence and potential benefits and risks of the various agricultural biotechnology applications among stakeholders. COMESA will take the necessary steps and initiatives to mobilize resources for continuous and strategic capacity building of Member States with limited capacity for risk assessment and regulation of GMOs so as to enable them competently participate in the regional risk-assessment framework and to take informed decisions at country-level.”
- “Member States shall enhance or put in place mechanisms for continuous and regular monitoring of GMO events in their territories and keep the COMESA Secretariat updated.”
- “Whether or not to plant GMOs, trade with GMOs, or accept food aid with GM content will be the sovereign decision of Member States.”

### Opportunities in the implementation of the COMESA biotechnology and biosafety policy

The crops for which biotech/GM commodity products are available, within the COMESA region are banana, cassava, sorghum, sweet potato and cotton. Those that are available outside the region are maize, soybean, cowpea and rice. These crops are relevant and important in terms of food security and agri-business development in the COMESA region. Their products can find their way into the region through adoption for cultivation, formal and informal trade, and food-aid assistance. In the short-run, this can complicate the regulatory process within countries. In the long run, without a collective regional approach, it is possible that the GM factor may rise to the level of trade disruption between/among Member States as a result of different policies (Belay et al., 2014). Therefore, focusing on uncompromised region-wide biosafety risk-assessment instruments that are complementary to national-level systems is important. The idea is straightforward; without infringing on national-level decisions, for a given GM-event, if one country conducts the risk-assessment properly it should not necessarily be repeated in all the Member States. Given that both biotech product testing and regulatory requirements are scientific-knowledge and resource intensive, a regional-level biosafety risk-assessment system will help in pooling scarce resources.

The novelty of a regional approach to biosafety considerations calls for concerted efforts among key partners to ensure success. Sustained political support is key in embracing and operationalizing the regional biosafety policies. This can be achieved through continuous generation of evidence-based data for informed decision-making and implementation of a focused communication and outreach strategy. Capacity building will be a major requirement in ensuring that all countries have functional biosafety systems in place while standard operating procedures and structures for implementing the policy will have to be instituted.

Most COMESA member countries have embarked on the development of national biosafety policies, laws and regulations. While a few countries have progressed to more advanced stages of developing functional biosafety systems, the majority are still at the initial stages of development. Countries will be encouraged to make use of the COMESA biosafety road map as they develop and strength their national biosafety frameworks. This will ensure complementarity and the desired interface with the regional biosafety decision-making arrangements.

In the long term, a regional approach to biosafety is expected to foster inter-country cooperation through the sharing of knowledge, expertise, experiences and resources. The COMESA biosafety implementation plan will guide implementation of the COMESA policy across the region. It will be the first initiative of the COMESA long-term program on biotechnology and biosafety.

### Conclusion and lesson learnt

The pivotal provisions included in the COMESA biosafety policy are as follows: (a) collective recognition of both the benefits and potential risks associated with GMOs in a case-by-case approach; (b) a regional-level and science-based biosafety risk-assessment mechanism, coupled with national-level decision-making; and (c) capacity building. The regional biosafety policy stands for sharing of information, resources, and expertise, and reducing redundancies and cost of biosafety regulations. The processes of its formulation and approval underwent intensive consultations with key stakeholders in an inclusive, participatory, and interactive manner.

There have been important lessons learnt during the policy formulation process that are still relevant when venturing into the implementation of the policy. Key among these are that biotechnology/biosafety issues of regional harmonization should be handled in a consultative, participatory, and inclusive manner. This is because given the controversies that surround the technology, the process of policy formulation was as important as the policy framework itself. Deliberations cutting across the entire life cycle of the RABESA project took place in 24 national and 4 regional workshops.

Regional harmonization of biosafety policies is both a technical and political process. It requires strong political will and commitment at various levels within Member States. The progress made and political-buy in realized so far could be attributed to the fact that the RABESA project has been a recurrent agenda item in various COMESA policy organ meetings. This reflects the good will that COMESA’s highest levels maintained.

National sovereignty is a fundamental and sensitive issue. The convergence and divergence between national and regional frameworks had to be clearly spelled out. The pertinent concerns need to be handled carefully to dispel fears that the regional process may infringe on, or override national interests and decision-making powers.

A policy on its own is insufficient to deliver desired changes to a society unless it is implemented. Awareness and outreach efforts need to be stepped up in order for countries to appropriate the benefits of a regional approach in biosafety decision-making. This necessitates the need for a focused and demand-driven communication strategy and implementation plan to ensure that credible evidence is delivered to target audiences in the formats best suited for them.

## Conflict of Interest Statement

The authors declare that the research was conducted in the absence of any commercial or financial relationships that could be construed as a potential conflict of interest.
